# Characterization and classification of Rare Disease Registries by using exploratory data analyses

**DOI:** 10.1186/1750-1172-9-S1-P4

**Published:** 2014-11-11

**Authors:** Alessio Coi, Michele Santoro, Michele Lipucci, Anna Maria Bianucci, Sabina Gainotti, Emanuela Mollo, Luciano Vittozzi, Domenica Taruscio, Fabrizio Bianchi

**Affiliations:** 1 Fondazione Toscana Gabriele Monasterio, Pisa, Italy; 2 Institute of Clinical Physiology, National Council of Research, Pisa, Italy; 3 European Organisation for Rare Diseases (EURORDIS), Paris, France; 4 Department of Pharmacy, University of Pisa, Italy; 5 National Centre for Rare Diseases, National Institute for Health, Rome, Italy

## 

European Commission and Patients Associations identify Registries as strategic instruments to improve knowledge in the field of Rare Diseases [1,2]. Interoperability between Rare Diseases Patient Registries (RDPR) is especially needed to support research activities, to validate therapeutic treatments and to plan public health actions. Because of the extreme variety of RDPR, a uniform and standardized way of collecting data and the identification of specific levels of connection between RDPR with similar aims is needed.

In this study, exploratory data analyses were applied to the EPIRARE (European Platform for Rare Diseases Registries) Registry Survey in order to generate a macro-classification and characterization of RDPR and to deepen different informative needs.

**Figure 1:**
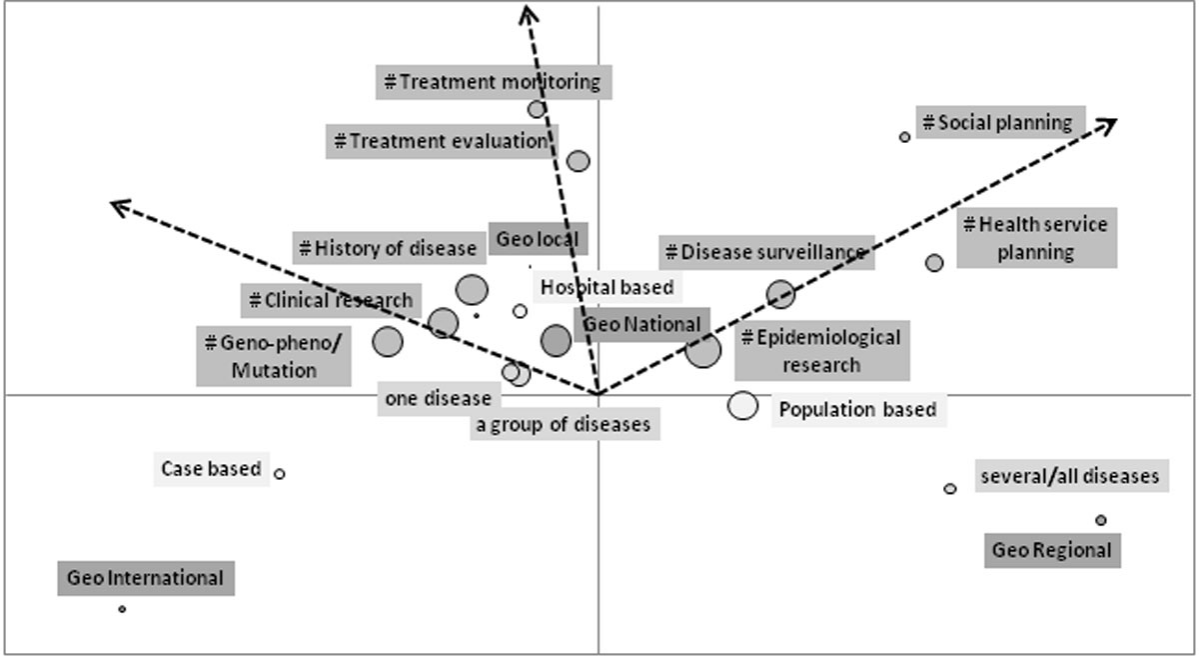
Factorial plan by MCA.

At first, a Multiple Correspondence Analysis (MCA) suggested associations between selected variables characterizing the structure of RDPR (Figure 1). Then, a Cluster analysis (CA) was developed using the declared “Aims” of each RDPR. CA confirmed the variable associations emerged by MCA and identified three groups defined as: Public Health (PHR), Clinical-Genetic Research (CGRR), and Treatment Registries (TR). Finally, the random forest (RF) method was applied to the Survey data, leading to six classification models endowed of good predictive power and thus confirming the reliability of considering three groups of RDPR. RF also identified several informative variables which allowed the characterization of the three categories of RDPR, defined by data of different nature and by different levels of diffusion (Table 1).

**Table 1:** Main characteristics of Clinical-Genetic Research, Treatment, and Public Health Registries according to the most informative variables emerged after the random forest method. Variables reported in the table characterize most of the registries of each class.

**Variables**	**Public Health Registries**	**Treatment Registries**	**Clinical-Genetic research Registries**
**Aims**	- epidemiologic research - disease surveillance - healthcare services planning	- treatment evaluation - treatment monitoring	- clinical research - genetic -natural history of the disease
**Collected data**	socio-demographic	- clinical - medications, devices and health services - genetic - family history - date of the patient death - patient-reported outcomes - anthropometric info	- clinical - genetic - family history
**Coding system**	ICD	No coding system or own code	No coding system or own code
**Services requested to a EU platform**	“Quality control systems”	“Facilitated access to useful data sources”	“Model documents”

These results, identifying different profiles of RDPR and specific informative needs, represent an informative support aimed at addressing the activities for the design of an European platform of Rare Diseases. Identification of informative cores could address the activities of a platform able to enhance the sharing of information between RDPR with common aims, but also to facilitate a coherent dialogue between RDPR with different profiles.

Guide to interpretation: the arrows indicate the directions of association among the aims; the dimension of the circles represents the frequency of the variable. The higher are the coordinate and the frequency of the variable, the more it contributes to the interpretation of the factorial axis; variables placed on the same direction are correlated.
